# *F8* Variants and Inhibitor Development in a Multiethnic Cohort of Nonsevere Haemophilia A

**DOI:** 10.1111/hae.70143

**Published:** 2025-10-10

**Authors:** Ming Y. Lim, Kristy Lee, Jill M. Johnsen, Nigel S. Key

**Affiliations:** ^1^ Department of Internal Medicine Division of Hematology and Hematologic Malignancies Salt Lake City Utah USA; ^2^ Department of Genetics University of North Carolina Chapel Hill North Carolina USA; ^3^ Department of Medicine University of Washington Seattle Washington USA; ^4^ Institute For Stem Cell and Regenerative Medicine University of Washington Seattle Washington USA; ^5^ Department of Medicine Division of Hematology and Blood Research Center University of North Carolina At Chapel Hill Chapel Hill North Carolina USA

**Keywords:** ethnicity, genotype, haemophilia A, racial groups, risk

## Abstract

**Background:**

Neutralising antibodies (inhibitors) against factor VIII can result in severe bleeding in persons with nonsevere haemophilia A (NSHA). The INSIGHT study of 1112 persons with NSHA in a predominantly White population identified 19 different *F8* missense variants that were associated with inhibitor development.

**Objective:**

To describe the *F8* variants and inhibitor development in persons with NSHA in a multiethnic cohort using the *My Life, Our Future* (MLOF) Research Repository and the American Thrombosis and Hemostasis Network dataset (ATHNdataset).

**Methods:**

The MLOF Research Repository and ATHNdataset were queried for demographic, clinical and genotyping data.

**Results:**

A total of 1805 persons with NSHA with at least one reportable *F8* variant and known inhibitor status were included in this study. Inhibitors were developed in 142 (7.9%) persons with NSHA. Inhibitor development occurred in seventy *F8* variants, of which the majority (*n* = 67, 95.7%) were missense variants. These 70 *F8 *variants were identified in a total of 1006 (55.7%) persons with NSHA. Race or ethnicity was not associated with inhibitors in persons with NSHA.

**Conclusion:**

The MLOF Research Repository identified additional *F8* variants where inhibitor development occurred in a multiethnic cohort of NSHA. Identification of these *F8* variants can inform both physicians and persons with NSHA to adopt measures to reduce the risk of inhibitor development.

## 1 Introduction

The development of neutralising antibodies, or inhibitors, against infused exogenous factor VIII (FVIII) represents a challenging complication in the treatment of haemophilia A. In persons with nonsevere haemophilia A (NSHA), inhibitor development is thought to be uncommon [1]. However, a landmark paper by Eckhardt et al. on behalf of the INSIGHT Study Group of 1112 persons with NSHA from 14 centres in Europe and Australia found that inhibitor development occurs throughout the lifetime in persons with NSHA, with a cumulative risk as high as 13.3% in individuals with >100 exposure days (ED) to FVIII [2]. Additionally, the INSIGHT study found that inhibitor development in persons with NSHA is associated with increased mortality [3] and thus deserves further evaluation.

The *F8* variant is an important risk factor for inhibitor development [4, 5]. In individuals with severe haemophilia A (SHA), the risk for inhibitor development is increased by variants that are more likely to be null, such as inversions, complex structural variants, or nonsense (stop‐gain) variants of the *F8* gene. In contrast, inhibitor development in NSHA is thought to be caused by *F8* missense variants that likely cause conformational changes within immunogenic domains of *F8* [6]. It has been demonstrated that among more than 150 different causative missense variants for NSHA, some relatively prevalent variants are associated with a high risk for inhibitor development [2, 6, 7]. The INSIGHT study found that among 214 different *F8* missense variants, 19 variants were associated with inhibitor development [2]. In certain rare variants (p.Asp2074Gly and p.Trp2229Cys*)*, the risk for inhibitor development at 20 ED (21.2% and 41.7%, respectively) parallels that seen in persons with SHA. Although it is not entirely clear why these particular variants carry an increased risk for inhibitors, the INSIGHT study was a first step towards the identification of high‐risk persons with NSHA based on their *F8* genotype.

Although the INSIGHT study was the largest study on persons with NSHA, it may not have captured other NSHA‐causing genotypes as it consisted of a predominantly White population (94.3%), with only 1.3% Black. Race and ethnicity are significantly associated with inhibitors in both haemophilia A and B [8] with the prevalence of FVIII inhibitors in the Black population about twice that of Whites [9]. Given this background, we set out to use the *My Life, Our Future* (MLOF) Research Repository to describe the *F8* variants and inhibitor development in a multiethnic cohort of NSHA.

## 2 Methods

### 2.1 Subjects and Study Design

The MLOF Research Repository contains de‐identified biologic samples and data from participants who enrolled in the MLOF genotyping project at haemophilia treatment centres (HTCs) across the United States (US). Details on participant enrolment, clinical data submitted, and *F8* variant screening and confirmation for the MLOF Research Repository can be found in Johnsen et al. [8]. All participants in MLOF also authorised for enrolment in the ATHNdataset. The ATHNdataset consists of a ‘limited dataset’ as defined under the Health Insurance Portability and Accountability Act to be free of protected health information. The ATHNdataset began enrolling alive persons with inherited bleeding disorders who have authorised sharing of their demographic and clinical information for research on 1 January 2010.

### 2.2 Data Collection and Definitions

MLOF eligibility included individuals with a clinical diagnosis of haemophilia A (for whom a reported FVIII level had to be <50%) and females at‐risk for or known to have a haemophilia A genotype (regardless of factor level). For this study, we included persons with NSHA who were categorised as moderate or mild haemophilia A using the lowest reported FVIII level of 1%–5% or >5%–40%, respectively [10, 11]. We excluded persons with FVIII levels of >40%. The datasets were queried on 31 December 2019 to extract the following: Demographics, haemophilia A severity, inhibitor status, *F8* variants, current or past infection with hepatitis B and/or hepatitis C virus, and presence of HIV infection. Demographics included age, gender, self‐classified race (White, Black, Asian and Others), and ethnicity (Hispanic and non‐Hispanic). The lowest reported FVIII level submitted during enrolment to the MLOF Research Repository was used to assign haemophilia severity.

Male individuals with one reportable *F8* variant consisting of either large structural change (>50 bp), frameshift, nonsense (stop‐gain), or canonical splicing (−1, −2, +1, or +2) variant for whom the lowest reported FVIII level was ≥1% were excluded from the study. It is thought that some of these MLOF participants classified as ‘nonsevere’ actually have SHA with an incorrectly reported lowest FVIII level [8]. For male individuals with one reportable *F8* variant consisting of either synonymous or noncanonical splicing variants for whom the lowest reported FVIII level was ≥1%, both the European Association for Hemophilia and Allied Disorders (EAHAD) Coagulation Factor VIII Variant Database [12] and the Centers for Disease Control and Prevention Hemophilia A Mutation Project (CHAMP) [13] were reviewed and those whose *F8* variants have been associated with SHA were also excluded. For male individuals with two reportable *F8* variants (*n* = 13), the variant predicted to be the most impactful was used to determine exclusion. Eleven male individuals had two missense variants, while two male individuals had one missense variant and one non‐disease‐causing synonymous variant [8]. No males with two reportable *F8* variants were excluded from this study. Female individuals who were heterozygous or compound heterozygous for any *F8* variants were included if their lowest reported FVIII level was ≥1 IU/dL, indicating a diagnosis of mild or moderate haemophilia A.

Inhibitor testing was performed locally at the discretion of each HTC and recorded in the ATHNdataset. There is no mandatory requirement for inhibitor test reporting in the ATHNdataset. A person with NSHA was classified as positive for inhibitor if a detectable FVIII inhibitor of ≥0.6 Bethesda inhibitor assay unit per mL (BU/mL) was reported at any time in the ATHNdataset and/or if an inhibitor was indicated clinically in the ‘Inhibitor Status’ field (i.e., active, history of, inactive). Otherwise, a person with NSHA was considered not to have had an inhibitor if all reported inhibitor titres were <0.6 BU/mL or below the threshold of detection, and/or if the ‘Inhibitor Status’ field indicated no inhibitor (i.e., no history of, ruled out). If the relevant Inhibitor status field was uninformative (i.e., evaluation pending, unknown, or blank) and no historical inhibitor titre data were available, the subject with NSHA was classified as Unknown.

For persons with NSHA with a positive inhibitor status, we also determined if they had a clinically relevant FVIII inhibitor [14]. The definition of a clinically relevant FVIII inhibitor was adapted from the INSIGHT study [2] and has been used in prior studies using the ATHNdataset [15, 16]. Briefly, a clinically relevant FVIII inhibitor was defined as having at least 2 inhibitor titres of ≥1.0 BU/mL at two different time points. Although the INSIGHT study also included persons with inhibitor titres between 0.6 and 1.0 BU/mL who had a decrease in factor VIII plasma level to at least 50% of the baseline level, or a reduced half‐life after factor VIII administration of <6 h, these data were not consistently available in the ATHNdataset and were not used in this study.

### 2.3 Statistical Analysis

We used descriptive statistical analyses. Categorical variables were expressed as frequencies and percentage values. The association of inhibitor status with race (White, Black, and Asian), ethnicity (Hispanic, and non‐Hispanic) and viral infections (Hepatitis B, Hepatitis C, and HIV infections) were performed using χ^2^ tests of independence with a predetermined level of significance of 0.05. Individuals of ‘other’ or ‘unknown’ race and ethnicity were excluded due to insufficient data. All statistical analyses were performed using Stata MP v16 (College Station, TX, USA).

## 3 Results and Discussion

### 3.1 Patient Characteristics

**FIGURE 1:**
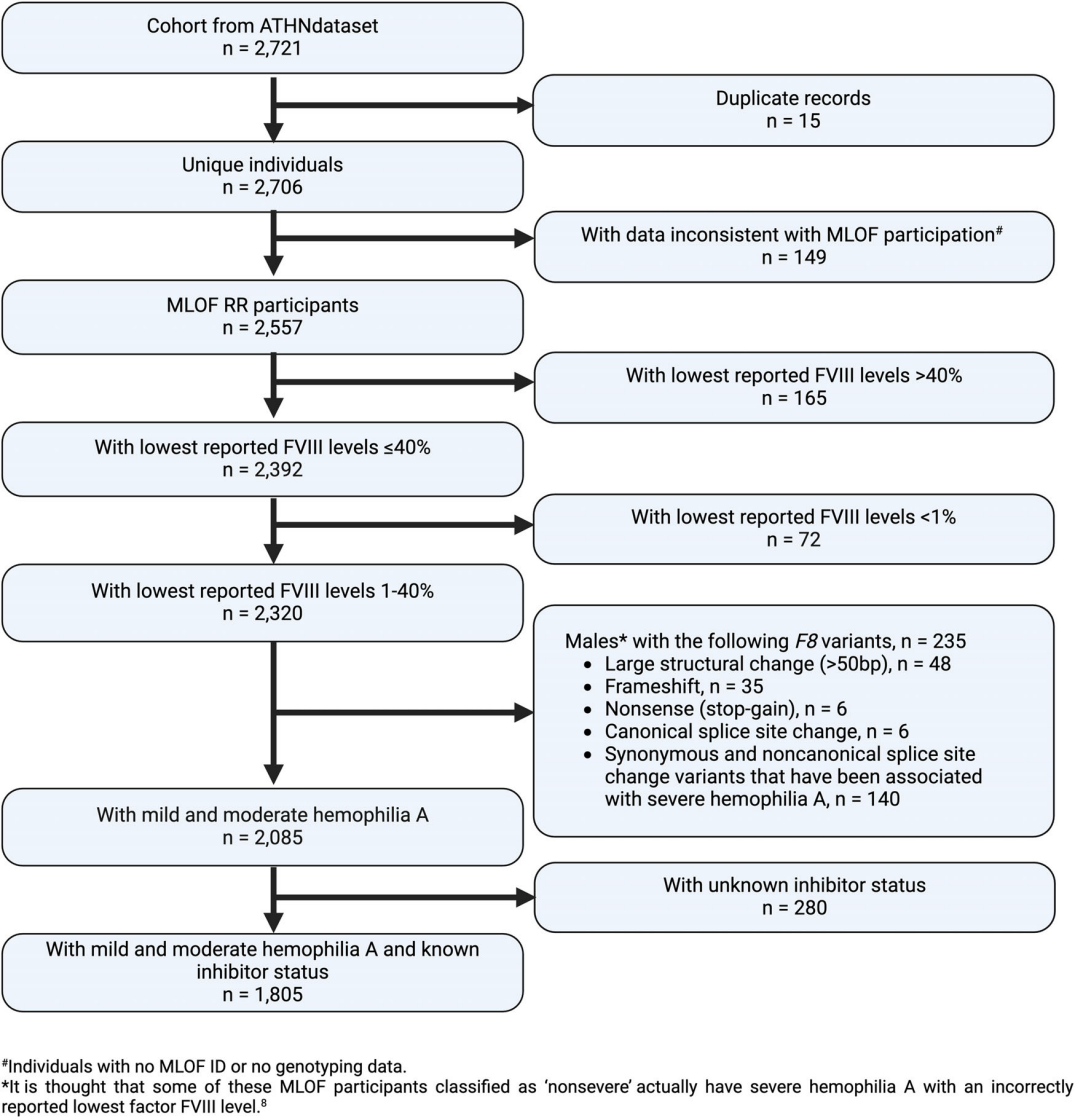
Flow chart describing data cleaning to identify persons with nonsevere haemophilia A by lowest reported FVIII levels with genotyping data and known inhibitor status.

A total of 1805 persons with NSHA with genotyping data and known inhibitor status were included in this study (Figure 1). The demographics and clinical characteristics of the NSHA cohort are shown in Table 1. Overall, the age distribution in the MLOF NSHA cohort was comparable with the NSHA data from the population of US HTCs from 2012 to 2022 [17].

**TABLE 1:** Demographics and clinical characteristics of the NSHA cohort (*n *= 1805).

	Overall (*n *= 1805)		No inhibitor (*n *= 1663)		Inhibitor (*n *= 142)	
Demographics	*N*	%	*N*	%	*N*	%
**Age (as of 2018)**						
<2 years	13	0.7	12	0.7	1	0.7
2 to 10	314	17.4	297	17.9	17	12.0
11 to 19	421	23.3	394	23.7	27	19.0
20 to 44	572	31.7	525	31.6	47	33.1
45 to 64	309	17.1	279	16.8	30	21.1
65+	157	8.7	141	8.5	16	11.3
Died	19	1.1	15	0.9	4	2.8
**Birth year**						
1921 to 1940	39	2.2	35	2.1	4	2.8
1941 to 1960	255	14.1	230	13.8	25	17.6
1961 to 1980	319	17.7	291	17.5	28	19.7
1981 to 2000	570	31.6	519	31.2	51	35.9
2001 to 2017	622	34.5	588	35.4	34	23.9
**Gender**						
Male	1593	88.3	1452	87.3	141	99.3
Female	212	11.7	211	12.7	1	0.7
**Race**						
White	1541	85.4	1417	85.2	124	87.3
Black	121	6.7	110	6.6	11	7.7
Asian	38	2.1	36	2.2	2	1.4
Other	56	3.1	55	3.3	1	0.7
Unknown	49	2.7	45	2.7	4	2.8
**Ethnicity**						
Hispanic	270	15.0	251	15.1	19	13.4
Non‐Hispanic	1519	84.2	1397	84.0	122	85.9
Unknown	16	0.9	15	0.9	1	0.7
**Severity**						
Mild	1194	66.1	1136	68.3	58	40.8
Moderate	611	33.9	527	31.7	84	59.2
**Hepatitis B**						
No	1776	98.4	1638	98.5	138	97.2
Yes	29	1.6	25	1.5	4	2.8
**Hepatitis C**						
No	1651	91.5	1528	91.9	123	86.6
Yes	154	8.5	135	8.1	19	13.4
**HIV**						
No	1764	97.7	1628	97.9	136	95.8
Yes	41	2.3	35	2.1	6	4.2

### 3.2 *F8* variants and Inhibitor Development

Inhibitors developed in 142 (7.9%) persons with NSHA (males, *n* = 141; females, *n* = 1). Inhibitor development occurred in seventy *F8* variants, of which the majority (*n* = 67, 95.7%) were missense variants (Table 2). These seventy *F8 *variants were identified in a total of 1006 (55.7%) persons with NSHA. The female individual with inhibitor had a baseline FVIII level of 30% and a heterozygous large structural change *F8* variant (c.[6429+?_6430‐?inv]).

**TABLE 2:** *F8* variants identified in persons with nonsevere haemophilia A and inhibitors.

Variant Impact (Variant Type)	HGVS cDNA	HGVS Protein	No. of persons with variant in overall cohort, *n* (%)^a^	No. of persons with inhibitors, *n* (%)^b^	No. of persons with clinically relevant FVIII inhibitors, *n*	Notes
Missense	c.80G>A	p.Gly27Asp	1 (0.1)	1 (100.0)		
Missense	c.311T>A	p.Val104Asp	9 (0.5)	1 (11.1)	1	
Missense	c.344T>C	p.Val115Ala	5 (0.3)	1 (20.0)		
Missense	c.403G>C	p.Asp135His	1 (0.1)	1 (100.0)		
Missense	c.490G>A	p.Gly164Ser	1 (0.1)	1 (100.0)	1	
Missense	c.544G>T	p.Asp182Tyr	8 (0.4)	1 (12.5)		
Missense	c.818A>G	p.Tyr273Cys	1 (0.1)	1 (100.0)		
Missense	c.871G>A	p.Glu291Lys	20 (1.1)	1 (5.0)		Reported in 17 hemizygous males and 3 heterozygous females
Missense	c.878A>G	p.His293Arg	1 (0.1)	1 (100.0)	1	
Missense	c.935T>C	p.Phe312Ser	46 (2.5)	3 (6.5)	2	Reported in 44 hemizygous males and 2 heterozygous females
Missense	c.1015A>G	p.Met339Val	5 (0.3)	1 (20.0)		
Missense	c.1172G>A	p.Arg391His	23 (1.3)	2 (8.7)	1	Reported in 21 hemizygous males and 2 heterozygous females
Missense	c.1312A>T	p.Ile438Phe	3 (0.2)	1 (33.3)		
Missense	c.1316G>T	p.Gly439Val	10 (0.6)	1 (10.0)	1	
Missense	c.1397G>A	p.Gly466Glu	1 (0.1)	1 (100.0)		
Missense	c.1475A>G	p.Tyr492Cys	3 (0.2)	1 (33.3)		
Missense	c.1478A>C	p.Asn493Thr	1 (0.1)	1 (100.0)		
Missense	c.1594T>G	p.Trp532Gly	1 (0.1)	1 (100.0)	1	
Missense	c.1636C>T	p.Arg546Trp	37 (2.0)	1 (2.7)		Reported in 33 hemizygous males and 4 heterozygous females
Missense	c.1648C>T	p.Arg550Cys	31 (1.7)	2 (6.5)	1	
Missense	c.1649G>A	p.Arg550His	14 (0.8)	3 (21.4)	1	Reported in 13 hemizygous males and 1 heterozygous female
Missense	c.1660A>G	p.Ser554Gly	58 (3.2)	4 (6.9)		Reported in 57 hemizygous males and 1 heterozygous female
Missense	c.1813T>C	p.Tyr605His	1 (0.1)	1 (100.0)		
Missense	c.1834C>T	p.Arg612Cys	103 (5.7)	12 (11.7)	5	Reported in 102 hemizygous males and 1 heterozygous female. The female has an additional causative variant (c.[5954delG])
Missense	c.2053G>A	p.Asp685Asn	1 (0.1)	1 (100.0)		
Missense	c.2087C>T	p.Thr696Ile	7 (0.4)	1 (14.3)		
Missense	c.2101A>G	p.Met701Val	2 (0.1)	1 (50.0)		
Missense	c.2167G>A	p.Ala723Thr	116 (6.4)	9 (7.8)		Reported in 110 hemizygous males and 6 heterozygous females. One female has an additional causative variant (c.[6066C>G])
Missense	c.5096A>C	p.Tyr1699Ser	4 (0.2)	3 (75.0)		
Missense	c.5096A>T	p.Tyr1699Phe	43 (2.4)	3 (7.0)		Reported in 40 hemizygous males and 3 heterozygous females
Missense	c.5122C>T	p.Arg1708Cys	7 (0.4)	2 (28.6)		
Missense	c.5399G>A	p.Arg1800His	28 (1.6)	1 (3.6)		Reported in 27 hemizygous males and 1 heterozygous female
Missense	c.5721C>G	p.Ser1907Arg	1 (0.1)	1 (100.0)		
Missense	c.5726A>G	p.Tyr1909Cys	1 (0.1)	1 (100.0)		
Missense	c.5822A>G	p.Asn1941Ser	46 (2.5)	6 (13.0)	3	Reported in 43 hemizygous males and 3 heterozygous females
Missense	c.5843T>C	p.Leu1948Pro	8 (0.4)	2 (25.0)	1	
Missense	c.5879G>A	p.Arg1960Gln	16 (0.9)	5 (31.3)		
Missense	c.6016G>A	p.Glu2006Lys	5 (0.3)	1 (20.0)		
Missense	c.6046C>T	p.Arg2016Trp	20 (1.1)	1 (5.0)		Reported in 19 hemizygous males and 1 heterozygous female
Missense	c.6113A>G	p.Asn2038Ser	10 (0.6)	2 (20.0)	1	Reported in 9 hemizygous males and 1 heterozygous female
Missense	c.6206T>C	p.Leu2069Pro	1 (0.1)	1 (100.0)		
Missense	c.6265T>C	p.Trp2089Arg	1 (0.1)	1 (100.0)		
Missense	c.6267G>T	p.Trp2089Cys	1 (0.1)	1 (100.0)		
Missense	c.6277G>C	p.Asp2093His	3 (0.2)	1 (33.3)	1	
Missense	c.6316C>G	p.Gln2106Glu	5 (0.3)	1 (20.0)	1	
Missense	c.6320G>A	p.Gly2107Asp	2 (0.1)	1 (50.0)		
Missense	c.6371A>G	p.Tyr2124Cys	15 (0.8)	4 (26.7)	2	Reported in 14 hemizygous males and 1 heterozygous female
Missense	c.6393G>T	p.Trp2131Cys	1 (0.1)	1 (100.0)		
Missense	c.6443A>G	p.Asn2148Ser	8 (0.4)	1 (12.5)		
Missense	c.6469A>G	p.Asn2157Asp	4 (0.2)	2 (50.0)		
Missense	c.6506G>A	p.Arg2169His	85 (4.7)	21 (24.7)	7	Reported in 82 hemizygous males and 3 heterozygous females
Missense	c.6518C>A	p.Thr2173Asn	3 (0.2)	1 (33.3)		
Missense	c.6533G>A	p.Arg2178His	7 (0.4)	1 (14.3)		
Missense	c.6533G>T	p.Arg2178Leu	15 (0.8)	1 (6.7)		Reported in 14 hemizygous males and 1heterozygous female
Missense	c.6544C>T	p.Arg2182Cys	6 (0.3)	1 (16.7)		Reported in 2 hemizygous males and 4 heterozygous females
Missense	c.6551A>C	p.Glu2184Ala	3 (0.2)	1 (33.3)		
Missense	c.6658G>C	p.Ala2220Pro	14 (0.8)	1 (7.1)		Reported in 13 hemizygous males and 1 heterozygous female
Missense	c.6679G>A	p.Ala2227Thr	8 (0.4)	2 (25.0)		
Missense	c.6683G>A	p.Arg2228Gln	3 (0.2)	1 (33.3)		
Missense	c.6715A>G	p.Arg2239Gly	3 (0.2)	1 (33.3)		
Missense	c.6752T>C	p.Val2251Ala	3 (0.2)	1 (33.3)		
Missense	c.6795G>T	p.Gln2265His	1 (0.1)	1 (100.0)		
Missense	c.6845C>T	p.Ser2282Phe	1 (0.1)	1 (100.0)		
Missense	c.6967C>G	p.Arg2323Gly	8 (0.4)	1 (12.5)		
Missense	c.6967C>T	p.Arg2323Cys	9 (0.5)	1 (11.1)		Reported in 8 hemizygous males and 1 heterozygous female
Missense	c.6968G>C	p.Arg2323Pro	5 (0.3)	1 (20.0)	1	
Missense	c.6977G>A	p.Arg2326Gln	18 (1.0)	3 (16.7)		
Splice site change	c.1903+5G>T		1 (0.1)	1 (100.0)		
Splice site change	c.5219+3A>G		10 (0.6)	2 (20.0)		Reported in 8 hemizygous males and 2 heterozygous females
Large structural change (>50 bp)	c.[6429+?_6430‐?inv]		63 (3.5)	1^c^ (1.6)		Reported in 63 heterozygous females, for which 3 females had additional causative variant (c.[1‐?_6429+?del]; c.[6430‐?_6900+?dup]; c.[1569G>T])
**Total**			1006 (55.7)	142	32	

^a^Percentage of the total cohort of persons with NSHA (*n* = 1805).

^b^Percentage of the number of persons with NSHA with the same *F8* variant.

^c^Heterozygous female.

Seven *F8* missense variants were reported in >40 persons with NSHA. Inhibitor development occurred in all seven *F8* missense variants: p.Ala723Thr (9/116, 7.8%), p.Arg612Cys (12/103, 11.7%), p.Arg2169His (21/85, 24.7%), p.Ser554Gly (4/58, 6.9%), p.Asn1941Ser (6/46, 13.0%), p.Phe312Ser (3/46, 6.5%), and p.Tyr1699Phe (3/43, 7.0%).

Thirty‐two persons with NSHA, comprising 18 *F8* missense variants, had a clinically relevant FVIII inhibitor (Table 2). The three most common *F8* missense variants observed in those with inhibitors and those with clinically relevant FVIII inhibitors were c.1834C>T (p.Arg612Cys), c.5822A>G (p.Asn1941Ser), and c.6506G>A (p.Arg2169His).

### 3.3 Association of Inhibitor Development and Race, Ethnicity, or Viral Infection

In persons with NSHA, a *χ* ^2^ test of independence of the three racial groups (White, Black and Asian) and inhibitor status found no association between race and inhibitors (Table S1). Similarly, there was no association between ethnicity and inhibitors. Inhibitors were associated with infection with hepatitis C (χ^2^ = 4.6426, *p*‐value = 0.031188), but not with hepatitis B and HIV infection.

## 4 Discussion

We describe the largest cohort of persons with NSHA and inhibitor development using the MLOF Research Repository and a uniform data collection tool – the ATHNdataset. This study identified 70 *F8* variants where inhibitor development occurred in a multiethnic cohort of NSHA. Of these 70 variants, six (p.Arg550Cys, p.Arg612Cys, p.Arg2169His, p.Arg2016Trp, p.Tyr2124Cys, p.Val2251Ala) were also identified in the INSIGHT study [2]. Overall, the MLOF cohort identified an additional 64 *F8* variants where inhibitor development occurred in our multiethnic cohort. Knowledge of inhibitor development occurring in these *F8* variants could help identify at‐risk individuals with NSHA based on their *F8* variant. Specifically, three of the most common *F8* variants that were seen in our study, namely p.Arg612Cys, p.Arg2169His, and p.Asn1941Ser had a rate of inhibitor development of >10%. Two of these, p.Arg612Cys and p.Arg2169His, were observed as well in the INSIGHT study and had a cumulative inhibitor risk of 18.3% and 12.2%, respectively, at 50 EDs [2] – a risk that approaches that of SHA. Identification of these at‐risk individuals can inform both physicians and persons with NSHA to adopt measures to reduce the risk of inhibitor development. These measures include the use of desmopressin or avoiding intensive courses of treatment with FVIII concentrates where possible.

In our study of NSHA with inhibitors, race or ethnicity was not associated with inhibitor development. This is in contrast to prior studies, including the MLOF parent study, which found that Black and Asian individuals, and those of Hispanic ethnicity, had significantly higher inhibitor rates than White individuals [8, 18, 19, 20, 21]. These prior studies all included SHA, which may account for the discrepant findings. It is also possible that the lack of association seen is due to insufficient power from the smaller number of NSHA individuals with inhibitors. Notably, this sub‐study of NSHA within the MLOF cohort had a lower prevalence of Black participants (6.7%) than the parent study (9.6%) [8], suggesting an under‐representation of minorities with NSHA in haemophilia clinical studies.

This study has several limitations. First, the classification of NSHA was based on the lowest recorded FVIII level at participating HTCs. This led to discrepancies in the classification of severity and was addressed by removing male individuals with null *F8* variants or variants that have been associated with SHA. Second, inhibitor data entry in the ATHNdataset was not mandatory for participation in MLOF, which resulted in about ∼13% of subjects with unknown inhibitor status who were excluded from this analysis. These individuals were likely negative for inhibitors, as those with positive inhibitor status usually require more medical attention and would likely have had their data entered into the ATHNdataset. Lastly, the number of EDs to FVIII concentrates and detailed description on the circumstances surrounding inhibitor development were not available in the ATHNdataset. This limitation highlights a critical need for data collection on number of EDs and other risk factors for inhibitor development to develop a personalised inhibitor risk stratification model based on *F8* variants.

## 5 Conclusion

The MLOF Research Repository offered a unique opportunity to uncover insights previously not possible, such as additional *F8* variants where inhibitor development occurred in a multiethnic cohort. Additionally, race and ethnicity were not associated with inhibitor development in persons with NSHA and inhibitors. Identifying persons with NSHA at‐risk for inhibitor development is important for the clinical management of this population.

## Author Contributions

Ming Y. Lim, Kristy Lee, Jill M. Johnsen, and Nigel S. Key participated in study methods design, data analysis, interpretation of results, and writing the manuscript.

## Ethics Statement

As the data obtained for this study were deidentified, this study was considered nonhuman subject research by the University of Utah institutional review board.

## Conflicts of Interest

M.Y.L. reports advisory board participation for Sanofi, Biomarin, Bayer, and Hema Biologics, and her institution has received research funding on her behalf from Sanofi. K.L. has no relevant disclosures. J.M.J. reports consulting for CSL Behring, Biomarin, Octapharma, and Takeda, and her institution has received research funding on her behalf from Octapharma. N.S.K. reports consulting for Pfizer, Centessa, and Novo Nordisk.

## Supporting information




**Supplemental Table 1**: Association of inhibitor development and race, ethnicity, or viral infection.

## Data Availability

The data that support the findings of this study are available on request from the corresponding author. The data are not publicly available due to privacy or ethical restrictions.
