# Supplementary material for: F8 Variants and Inhibitor Development in a Multiethnic Cohort of Nonsevere Haemophilia A
Source: Haemophilia. 2025 Oct 10;31(6):1217–25. doi: 10.1111/hae.70143 (PMC12612360; doi:10.1111/hae.70143)
Supplement: Supplementary file 1 — Supplemental Table 1: Association of inhibitor development and race, ethnicity, or viral infection. [file HAE-31-1217-s001.docx]

Supplemental Table 1. Association of inhibitor development and race, ethnicity, or viral infection

| **Characteristics** | **Non-inhibitor (n)** | **Inhibitor (n)** | **χ2** | ***p*-value** |
| --- | --- | --- | --- | --- |
| **Race** |  |  | 0.5751 | 0.750099 |
| White | 1417 | 124 |  |  |
| Black | 110 | 11 |  |  |
| Asian | 36 | 2 |  |  |
|  |  |  |  |  |
| **Ethnicity** |  |  | 0.3123 | 0.576251 |
| Hispanic | 251 | 19 |  |  |
| Non-Hispanic | 1397 | 122 |  |  |
|  |  |  |  |  |
| **Hepatitis B** |  |  | 1.428 | 0.232085 |
| No | 1638 | 138 |  |  |
| Yes | 25 | 4 |  |  |
|  |  |  |  |  |
| **Hepatitis C** |  |  | 4.6426 | **0.031188** |
| No | 1528 | 123 |  |  |
| Yes | 135 | 19 |  |  |
|  |  |  |  |  |
| **HIV** |  |  | 2.6506 | 0.103511 |
| No | 1628 | 136 |  |  |
| Yes | 35 | 6 |  |  |

* Level of significance of .05
